# Japanese Spotted Fever Complicated With Life-Threatening Ventricular Tachycardia and Acute Cardiac Damage

**DOI:** 10.7759/cureus.18408

**Published:** 2021-09-30

**Authors:** Daisuke Himeji, Shota Nasu, Gen-ichi Tanaka, Kouchi Masuda, Ryota Matsuura

**Affiliations:** 1 Department of Internal Medicine, Miyazaki Prefectural Miyazaki Hospital, Miyazaki, JPN; 2 Department of Psychiatry, Miyazaki Prefectural Miyazaki Hospital, Miyazaki, JPN; 3 Department of Cardiology, Miyazaki Prefectural Miyazaki Hospital, Miyazaki, JPN; 4 Department of Hemovascular Medicine and Artificial Organs, Faculty of Medicine, University of Miyazaki, Miyazaki, JPN

**Keywords:** rickettsial diseases, takotsubo cardiomyopathy, acute cardiac damage, ventricular tachycardia, japanese spotted fever

## Abstract

A 67-year-old man with high-grade fever and systemic erythema visited our hospital. Based on his symptoms and history of outdoor activities, we considered the possibility of rickettsial diseases, especially Japanese spotted fever (JSF). He was treated with antibiotics. After hospitalization, the patient complained of palpitations, and electrocardiography revealed ventricular tachycardia (VT). He was successfully treated with electrical defibrillation. Moreover, echocardiography showed decreased wall motion at the apex. However, coronary angiography showed no coronary artery-related stenosis. JSF was confirmed via polymerase chain reaction using a biopsy sample of the erythema. Subsequently, the patient was discharged without complications. To our knowledge, this is the first reported case of JSF complicated with VT and acute cardiac damage.

## Introduction

Japanese spotted fever (JSF) is a severe rickettsiosis caused by *Rickettsia japonica* [1]. Recently, reported cases of JSF in Japan have increased from 66 in 2004 to 337 in 2017, and the geographic area of infection has expanded [2]. In addition, 31 deaths have been reported between April 1994 and the end of 2018 (case fatality rate, 1.1%). In particular, 13 deaths were reported in 2019 alone (case fatality rate 4.1%, 13/318), therefore, JSF is a public health concern [2]. Severe or fatal cases of JSF complicated with disseminated intravascular coagulation, multiple organ failure, meningoencephalitis, central nervous system disorders, and acute respiratory distress syndrome have been reported previously [3].

However, cases of JSF complicated with life-threatening arrhythmias and acute cardiac damage have not been reported. In addition, only one case of death from Takotsubo cardiomyopathy has been reported [3] but the details of the case were not provided. Here we report the case of JSF with life-threatening arrhythmia, ventricular tachycardia (VT), and acute cardiac damage.

## Case presentation

**Figure 1:**
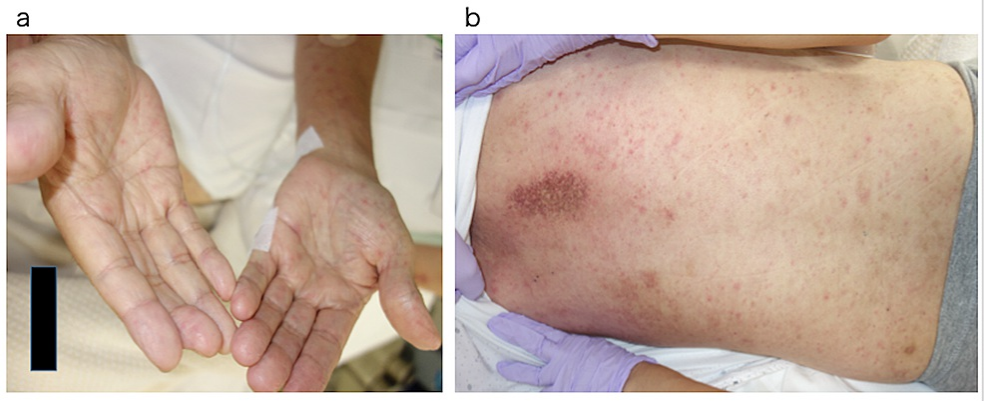
(a) Erythema on the palms. (b) Erythema on the trunk.

**Figure 2:**
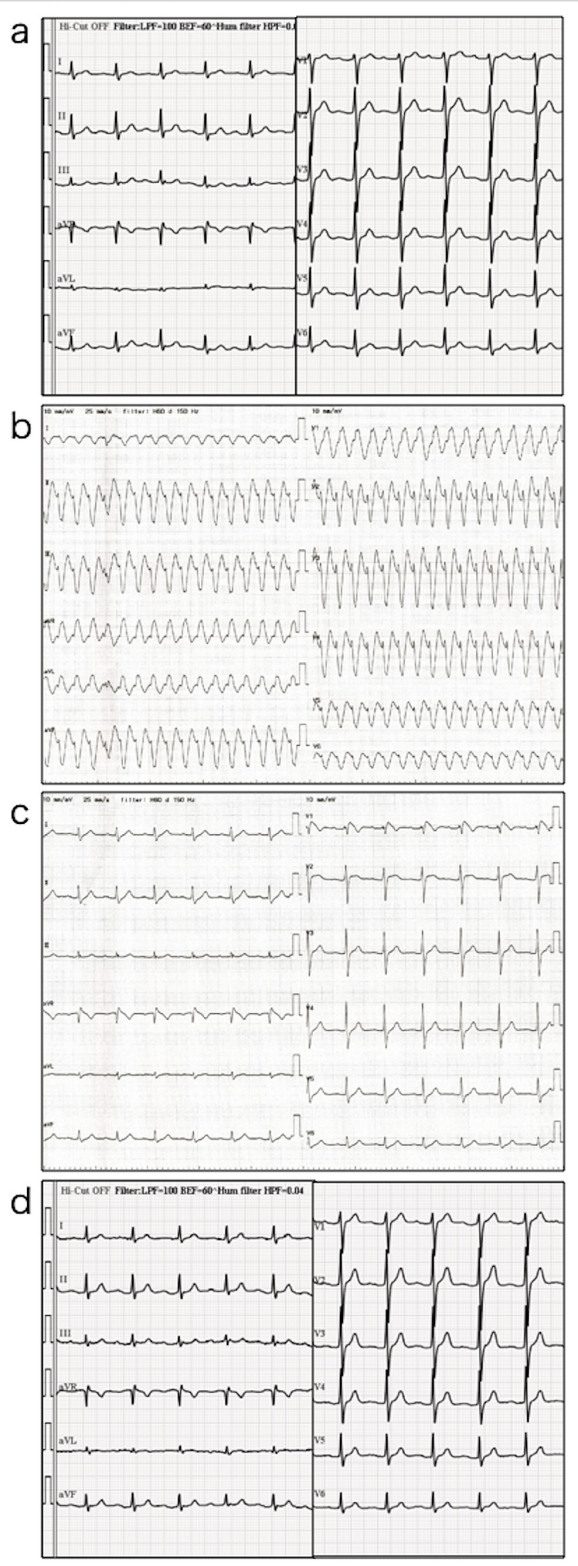
Time course and electrocardiography findings: (a) one year before admission; (b) at the onset of ventricular tachycardia (VT); (c) immediately after recovery from VT; (d) one year after VT.

A 67-year-old man, who works as a farmer, visited our hospital with a six-day history of high-grade fever and systemic erythema. Nausea and vomiting reportedly occurred the day before the visit. Three years ago, he was diagnosed with lung squamous carcinoma, cT1bN2M0, and was treated with concurrent chemoradiotherapy with cisplatin and Tegafur/Gimeracil/Oteracil to which he achieved a partial response. Two years later, a metastatic tumor developed in the right lower lung; thus, he underwent repeated chemotherapy using carboplatin and paclitaxel. The lesion was then stabilized after four cycles of chemotherapy. His vital signs were as follows: temperature, 39.6°C; blood pressure, 115/79 mm Hg; pulse rate, 117 beats/min; respiratory rate, 20 breaths/min; and O2 saturation, 96% (room air). Furthermore, erythematous blotches were observed over his whole body, especially on the palms and soles (Figure 1); however, he did not complain of itching. Neither lymphadenopathy nor hepatosplenomegaly was noted. Hemogram results revealed a normal leukocyte count of 9,620/μL, and liver function tests were normal. However, lactate dehydrogenase(268 U/l), blood urea nitrogen(28.1 mg/dL), and creatinine levels (1.59 mg/dL) were elevated, and the C-reactive protein level was 21.2 mg/dL. Blood cultures revealed no growth. Electrocardiogram (ECG) performed one year before admission was normal (Figure 2a).

We considered Rickettsial diseases, especially JSF, as differential diagnoses on the basis of his fever, characteristic erythematous eruption, and his history of outdoor activities. Hence, he was tentatively diagnosed with JSF and treated with minocycline (MINO) 100 mg twice a day and levofloxacin (LVFX) 500 mg intravenously.

**Figure 3:**
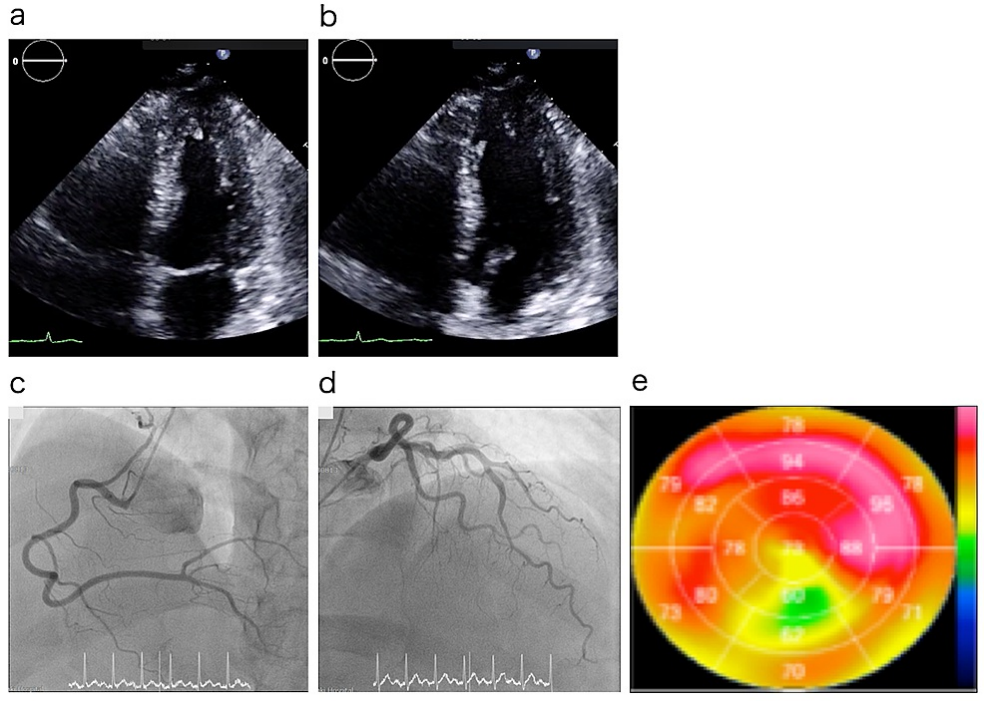
Transthoracic echocardiogram, coronary angiography and 123I- metaiodobenzylguanidine (MIBG) myocardial scintigraphy. Transthoracic echocardiogram performed immediately after the recovery from ventricular tachycardia showing hypokinesia of the left ventricular apex. Apical approach, four-chamber view: (a) systolic phase; (b) diastolic phase. Coronary angiography (CAG) performed three days after ventricular tachycardia and 123I-metaiodobenzylguanidine (MIBG) myocardial scintigraphy performed 10 days after discharge. CAG revealed no cardiac contractility and no coronary arterial stenosis: (c) right coronary artery; (d) left coronary artery. (e) Bull’s eye image. MIBG accumulation decreased mainly in the apex.

In the morning of the following day, the patient complained of fever and chills, as well as palpitations; thus, electrocardiography was performed, which showed VT (Figure 2b). He was conscious during this time, and his vital signs were as follows: temperature, 38.6°C; blood pressure, 112/57 mmHg; and pulse rate, 172 beats/min. He was then treated with intravenous injections of lidocaine hydrochloride, propranolol hydrochloride, and amiodarone hydrochloride; however, his sinus rhythm failed to resume. Thus, he was sedated using midazolam injection and underwent electrical cardioversion (100 J). Upon recovery, his blood pressure was 110/79 mmHg and pulse rate was 102 beats/min. ECG showed an incomplete right bundle block, ST elevation in V1, and no QT prolongation (Figure 2c). Echocardiography performed immediately after recovery revealed decreased wall motion at the apex (Figure 3a and 3b); thus, we suspected acute cardiac damage resembling Takotsubo cardiomyopathy.

Further, five hours after this event, his body temperature increased to 39.2°C, his systolic blood pressure decreased to 70 mmHg, and he was in warm shock. He was then treated with fluid resuscitation and 0.04γ of noradrenaline; eventually, his blood pressure increased, and he recovered from the state of shock. To eliminate ischemic heart disease, we repeatedly measured myocardial deviation enzymes, such as creatine kinase and troponin T, but no increase was observed.

Thereafter, the circulation gradually became stable. Antibacterial treatments with MINO and LVFX were continued for two weeks. Furthermore, five days after admission, he was diagnosed with JSF via polymerase chain reaction using the biopsy sample of erythema. Coronary angiography performed seven days after admission revealed no cardiac contractility and no stenosis of the coronary arteries (Figs. 3c and 3d). Thus, we concluded that the patient’s condition did not involve ischemic heart disease. Intravenous MINO and LVFX were administered for seven days, then changed to oral administration and administered for seven days. He was discharged 10 days after admission without any problems.

Further, 10 days after discharge, 123^I^- metaiodobenzylguanidine (MIBG) myocardial scintigraphy performed revealed a decrease in MIBG accumulation mainly in the apex (Figure 3e). These findings supported the diagnosis of acute cardiac damage resembling Takotsubo cardiomyopathy, and one year later, his ECG findings showed full recovery (Figure 2d). Appropriate written informed consent was obtained for publication of this case report and accompanying images.

## Discussion

To the best of our knowledge, this is the first case of JSF complicated with life-threatening arrhythmia, VT, and acute cardiac damage that resembles Takotsubo cardiomyopathy. On the basis of ECG findings, echocardiography, and MIBG myocardial scintigraphy, we postulated that the VT and acute cardiac damage were associated with the JSF infection.

Differentiation from septic cardiomyopathy is challenging; however, echocardiogram and myocardial scintigraphy findings suggested acute cardiac damage that resembles Takotsubo cardiomyopathy in this case. Stress cardiomyopathy, otherwise known as Takotsubo cardiomyopathy, is diagnosed on the basis of clinical presentation, a typical cardiac imaging pattern of transient myocardial wall motion abnormalities extending beyond the territory of one coronary vessel, and normal coronary arteries on angiography [4]. Takotsubo cardiomyopathy is generally considered a disease with good prognosis that completely recovers within days to weeks [4]. However, acute VT/ventricular fibrillation rate associated with Takotsubo cardiomyopathy is reported to be 1.5%-9% [5,6]. Cases of Takotsubo cardiomyopathy with in-hospital death, cardiac failure, and sudden death during acute phase have also been reported [7]. A possible hypothesis is that our patient may have developed acute cardiac damage that resembles Takotsubo cardiomyopathy from VT because no preceding emotional stress or symptoms specific to Takotsubo cardiomyopathy were noted. However, in many previous reports, VT developed during the course of Takotsubo cardiomyopathy. Therefore, whether VT or acute cardiac damage resembling Takotsubo cardiomyopathy occurred first in this patient remains unclear. The patient’s ECG had not been monitored until palpitations occurred after hospitalization, and no echocardiogram was performed; thus, the onset of the acute cardiac damage remains unknown. Considering that JSF can be associated with severe arrhythmias and cardiomyopathy, it is recommended that ECG monitoring is performed during hospitalization.

Takotsubo cardiomyopathy is generally related to physical or emotional stress. However, recently, there have been reports of Takotsubo cardiomyopathy associated with infection and sepsis [8]. Reportedly, hypercytokinemia is also observed in JSF [9]; hence, catecholamine release due to JSF-induced hypercytokinemia may lead to the onset of life-threatening arrhythmia and acute cardiac damage that resembles Takotsubo cardiomyopathy.

Acute cardiac damage, including Takotsubo cardiomyopathy, has rarely been associated with rickettsial disease [3]. Only three cases of myocardial disease in Rocky Mountain spotted fever and JSF have been reported [3,10,11]. In addition, only one report of a fatal case of JSF and myocardial injury is known, but details of the case were not described. Myocardial damage appears to be a rare complication of rickettsial infection, but once it occurs, it can lead to death, particularly in severe cases; thus, careful evaluation and monitoring are required. Additionally, collection of data on cases related to myocardial injury and the examination of inducers are necessary.

## Conclusions

Recently, 31 deaths related to JSF infection have been reported between April 1994 and the end of 2018 (case fatality rate, 1.1%). Therefore, JSF is a public health concern. However, cases of JSF complicated with life-threatening arrhythmias and acute cardiac damage have not been reported. To our knowledge, this is the first reported case of JSF complicated with life-threatening VT and acute cardiac damage that resembles Takotsubo cardiomyopathy. Considering that JSF can be associated with severe arrhythmias and acute cardiac damage, it is recommended that the ECG is monitored during hospitalization.
